# Supplementary material for: Regional quantification of cardiac metabolism with hyperpolarized [1-13C]-pyruvate CMR evaluated in an oral glucose challenge
Source: J Cardiovasc Magn Reson. 2023 Dec 14;25:77. doi: 10.1186/s12968-023-00972-7 (PMC10720165; doi:10.1186/s12968-023-00972-7)
Supplement: Supplementary file 1 — Additional file 1: Figure S1. Point spread functions for the 22 ms long single-shot spiral readout trajectory, using the average T2* values reported from Ma J, Chen J, Reed GD, et al. Cardiac measurement of hyperpolarized 13C metabolites using metabolite-selective multi-echo spiral imaging. Magnetic Resonance in Medicine. https://doi.org/10.1002/mrm.28796 (values noted in the figure) of T2* = 119 ms (pyruvate), T2* = 43 ms (lactate), and T2* = 64 ms (bicarbonate), compared to the ideal point spread function (T2* = “Inf”). These values were measured at the same field strength (3T) and from the same scanner vendor (GE Healthcare) as our study. Based on this, we estimated relative full-width half-maximum (FWHM) for pyruvate = 1.02, lactate = 1.06, and bicarbonate = 1.04, corresponding to effective resolutions of 6.1 mm for pyruvate, 12.7 mm for lactate, and 12.5 mm for bicarbonate in our studies. Figure S2. Examples of coil-corrected HP 13C area under the curve metabolite maps in healthy volunteer subjects (subjects HV6, left, and HV7, right). Note the coil-corrected maps are masked based on a signal threshold to reduce visualization of noise amplification. Table S1. Regional wall LV thickness, measured from Cine CMR, in all subjects that were included in the analysis. Table S2. Quantifications of pyruvate dehydrogenase flux from HP 13C-pyruvate MRI, measured across several ROIs in all subjects that were included in the analysis. Table S3. Quantifications of lactate dehydrogenase flux from HP 13C-pyruvate MRI, measured across several regions of interest in all subjects that were included in the analysis. [file 12968_2023_972_MOESM1_ESM.docx]

**“Regional quantification of cardiac metabolism with hyperpolarized [1-13C]-pyruvate MRI evaluated in an oral glucose challenge”**

**Additional Information**

*
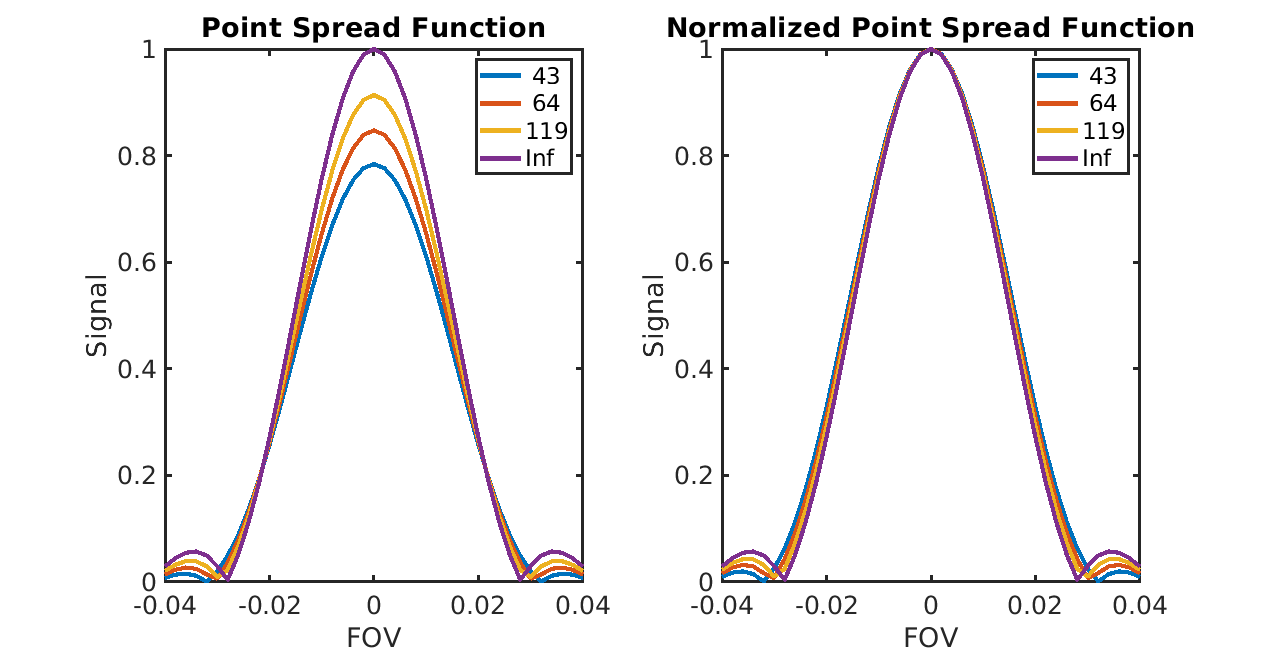
* **Figure S1:** Point spread functions for the 22 ms long single-shot spiral readout trajectory, using the average T2* values reported from Ma J, Chen J, Reed GD, et al. Cardiac measurement of hyperpolarized 13C metabolites using metabolite-selective multi-echo spiral imaging. Magnetic Resonance in Medicine. https://doi.org/10.1002/mrm.28796 (values noted in the figure) of T2* = 119 ms (pyruvate), T2* = 43 ms (lactate), and T2* = 64 ms (bicarbonate), compared to the ideal point spread function (T2* = “Inf”). These values were measured at the same field strength (3T) and from the same scanner vendor (GE Healthcare) as our study. Based on this, we estimated relative full-width half-maximum (FWHM) for pyruvate = 1.02, lactate = 1.06, and bicarbonate = 1.04, corresponding to effective resolutions of 6.1 mm for pyruvate, 12.7 mm for lactate, and 12.5 mm for bicarbonate in our studies.

**
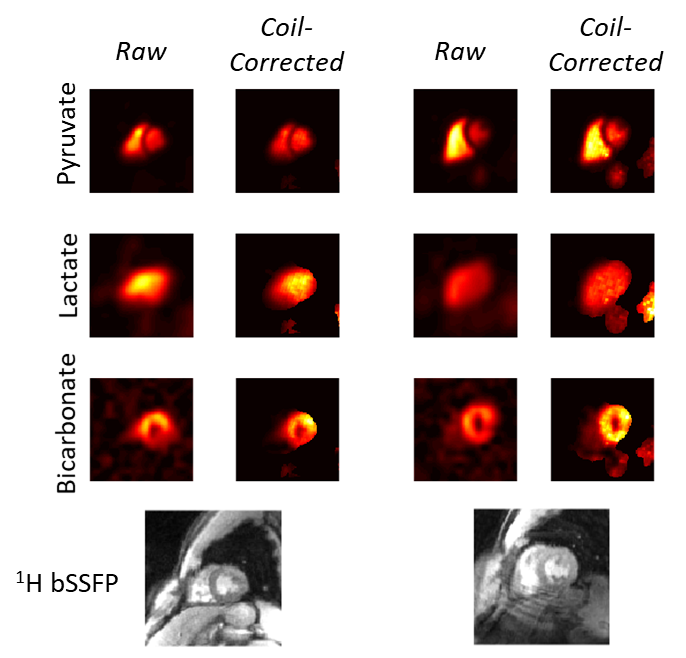
**

**Figure S2:** Examples of coil-corrected HP ^13^C area under the curve metabolite maps in healthy volunteer subjects (subjects HV6, left, and HV7, right). Note the coil-corrected maps are masked based on a signal threshold to reduce visualization of noise amplification.

| **Subject ID** | **Basal Septum**  **[cm]** | **Mid Septum**  **[cm]** | **Basal Anterior [cm]** | **Mid**  **Anterior**  **[cm]** | **Basal**  **Lateral**  **[cm]** | **Mid**  **Lateral**  **[cm]** | **Apex**  **[cm]** | **Inferior**  **[cm]** |
| --- | --- | --- | --- | --- | --- | --- | --- | --- |
| **HV1** | 8.3 | 5.1 | 5.2 | 4.6 | 5.0 | 4.9 | 3.7 | 3.7 |
| **HV3** | 6.8 | 6.2 | 5.8 | 4.6 | 6.3 | 5.7 | 5.2 | 4.9 |
| **HV4** | 7.6 | 6.2 | 5.5 | 4.3 | 6.2 | 6.1 | 4.5 | 4.1 |
| **HV6** | 8.0 | 7.6 | 6.3 | 4.6 | 6.2 | 6.2 | 6.2 | 3.6 |
| **HV7** | 7.6 | 8.0 | 6.0 | 5.6 | 7.1 | 5.8 | 5.2 | 3.9 |

**Table S1**: Regional wall LV thickness, measured from Cine CMR, in all subjects that were included in the analysis.

| **Subject ID** | **ROI** | **^13^C-bicarbonate /^13^C-pyruvate ratio** | | **k_PB_ [s^-1^]** | |
| --- | --- | --- | --- | --- | --- |
|  |  | Fasting | Fed | Fasting | Fed |
| **HV1** | Septum | 0.014 | 0.042 | 0.0010 | 0.0032 |
|  | Global LV | 0.020 | 0.067 | 0.0015 | 0.0051 |
| **HV3** | Septum | 0.013 | 0.033 | 0.0007 | 0.0026 |
|  | Global LV | 0.018 | 0.047 | 0.0011 | 0.0036 |
| **HV4** | Septum | 0.031 | 0.026 | 0.0019 | 0.0015 |
|  | Global LV | 0.040 | 0.032 | 0.0024 | 0.0020 |
| **HV6** | Septum | 0.016 | 0.038 | 0.0014 | 0.0028 |
|  | Global LV | 0.017 | 0.050 | 0.0012 | 0.0038 |
| **HV7** | Septum | 0.009 | 0.061 | 0.0004 | 0.0039 |
|  | Global LV | 0.014 | 0.090 | 0.0006 | 0.0061 |

**Table S2**: Quantifications of pyruvate dehydrogenase flux from HP ^13^C-pyruvate MRI, measured across several ROIs in all subjects that were included in the analysis.

| **Subject ID** | **ROI** | **^13^C-lactate /^13^C-pyruvate ratio** | | **k_PL_ [s^-1^]** | |
| --- | --- | --- | --- | --- | --- |
|  |  | Fasting | Fed | Fasting | Fed |
| **HV1** | Septum | 0.213 | 0.252 | 0.0115 | 0.0128 |
|  | Global LV | 0.255 | 0.311 | 0.0135 | 0.0160 |
| **HV3** | Septum | 0.238 | 0.412 | 0.0120 | 0.0214 |
|  | Global LV | 0.310 | 0.470 | 0.0153 | 0.0243 |
| **HV4** | Septum | 0.347 | 0.416 | 0.0148 | 0.0197 |
|  | Global LV | 0.363 | 0.454 | 0.0160 | 0.0215 |
| **HV6** | Septum | 0.303 | 0.338 | 0.0189 | 0.0184 |
|  | Global LV | 0.300 | 0.365 | 0.0169 | 0.0189 |
| **HV7** | Septum | 0.208 | 0.256 | 0.0086 | 0.0117 |
|  | Global LV | 0.234 | 0.328 | 0.0097 | 0.0161 |

**Table S3**: Quantifications of lactate dehydrogenase flux from HP ^13^C-pyruvate MRI, measured across several regions of interest in all subjects that were included in the analysis.
